# A Single-Handed Operation

**Published:** 1899-08-26

**Authors:** 


### A SINGLE-HANDED OPERATION.

The evidence given at an inquest recently held on a man who died at the Ipswich and East Suffolk Hospital after undergoing an operation should serve to draw attention to certain customs which we are afraid are by no means confined to that institution. It appears that the deceased, a labourer, 45 years of age, living at Somersham, went up to Ipswich in order to have a piece of diseased bone removed from the great toe. The operation was done by the assistant house surgeon, who took the patient into the operating-room, and, with the assistance of the senior sister, put him under chloroform and removed the bone. The operation was successfully performed, but on its conclusion the patient assumed the upright position, and vomited a good deal, and soon afterwards, while still on the table, he died. The sister stated that she dropped some chloroform on the cloth when the deceased moved during the progress of the operation. She added that she was always present at operations, that it was the custom to have a nurse present as well, and that the reason why they did not on this occasion have another nurse was that the outpatients were being sent away at the time. It appeared from the evidence of the senior house surgeon that he was in the house, although he was not present at the operation. Now, we would be the last to suggest that the benefits of anæsthesia are to be confined to those cases alone in which an anæsthetist can be employed. We are quite aware that it is held by many that it is as much as one man can do to administer an anæsthetic, but circumstances must often occur in which, if chloroform is not to be given unless it is administered by a qualified helper, the patient must go without it, and the exigencies of country practice are such that we should hesitate to lay down any hard-and-fast rule on such a subject. But in a hospital of considerable size, with two house surgeons, the matter is very different. Moreover, other things came out in the evidence which seem to show that at the hospital in question operations are hardly regarded with the gravity appropriate to cases in which anæsthetics have to be administered. According to the evidence of the junior house surgeon, as reported, he frequently performed operations under anæsthetics without any member of the medical staff being present; he had frequently, with the assistance of one sister, performed operations under chloroform; and neither the senior house surgeon nor any of the medical staff saw the deceased at the hospital. These are statements which we think call for the serious consideration of the board of management. Many other things came out in regard to this particular case to which we do not refer, because we are willing to believe that the house surgeon believed that he was acting in accordance with the custom of the place. But we distinctly think that it is wrong that in a hospital in which there are two house surgeons it should be a custom for the junior to consider himself at liberty to perform operations, even small ones, with the assistance of one sister, and on cases which have not only not been seen by any member of the staff, but not even by his own colleague.

